# Dr. Benjamin Moody

**Published:** 1914-10

**Authors:** 


Dr. Benjamin Moody, Geneva Medical College, 1868, Jefferson, 1869, died at his home in Mansfield, Pa., June 24, aged 73. He was a veteran of the Civil War.

